# Study of Grain Growth in a Ni-Based Superalloy by Experiments and Cellular Automaton Model

**DOI:** 10.3390/ma14226922

**Published:** 2021-11-16

**Authors:** Yan-Xing Liu, Zhi-Jiang Ke, Run-Hua Li, Ju-Qing Song, Jing-Jing Ruan

**Affiliations:** 1 Neutron Scattering Technical Engineering Research Center, School of Mechanical Engineering, Dongguan University of Technology, Dongguan 523808, China; kzj970916@163.com (Z.-J.K.); lirunhua_2021@163.com (R.-H.L.); songjuqing_shanxi@163.com (J.-Q.S.); 2 Guangdong-Hong Kong-Macao Joint Laboratory for Neutron Scattering Science and Technology, Dongguan University of Technology, Dongguan 523808, China; 3 Institute for Advanced Studies in Precision Materials, Yantai University, Yantai 264005, China

**Keywords:** grain growth, cellular automaton, Ni-based superalloy

## Abstract

The grain growth behavior in a typical Ni-based superalloy was investigated using isothermal heat treatment experiments over a holding temperature range of 1353–1473 K. The experimental results showed that the grain structure continuously coarsened as the holding time and holding temperature increased during heat treatment. A classical parabolic grain growth model was used to explore the mechanism of grain growth under experimental conditions. The grain growth exponent was found to be slightly above 2. This indicates that the current grain growth in the studied superalloy is mainly governed by grain boundary migration with a minor pinning effect from the precipitates. Then, the grain growth in the studied superalloy during isothermal heat treatment was modelled by a cellular automaton (CA) with deterministic state switch rules. The microscale kinetics of grain growth is described by the correlation between the moving velocity and curvature of the grain boundary. The local grain boundary curvature is well evaluated by a template disk method. The grain boundary mobility was found to increase with increasing temperature. The relationship between the grain boundary mobility and temperature has been established. The developed CA model is capable of capturing the dependence of the grain size on the holding time under different holding temperatures.

## 1. Introduction

The mechanical performances of components are sensitive to their microstructures, especially the grain size [1,2,3,4,5]. For instance, grain refinement can improve the strength and ductility of components at the same time by activating more slip systems [6,7]. This renders the control of grain size an important task. Ni-based superalloys have wide applications in high-temperature components, such as aircraft engines and gas turbines [8,9,10,11,12]. Thus, the study of grain growth behavior in Ni-based superalloys is of interest to many researchers [13,14,15,16,17,18]. Ruan et al. [13] found that grain growth in IN718 can be retarded by NbC and TiN phases due to Ostwald ripening. Lee et al. [14] found that the grain boundary structure can become rough at high temperatures, and it has a significant influence on the grain growth in a model Ni-based superalloy. Song and Aindow [15] conducted annealing experiments to study the grain growth in a model Ni-based superalloy and found that the pinning effect of the second-phase particles can lead to grain growth stagnation. Aoki et al. [16] analyzed the effects of strain on the grain size evolution in IN718 during post heat treatment. Tian et al. [17] simulated the grain size evolution in a Ni-based superalloy and found that a dual microstructure can be obtained by controlling the treating temperatures. Collins et al. [18] studied the grain growth behavior of RR1000 and found that there is a grain size limit for grain growth.

Most of the grain size models within the above research are of a classical parabolic type. These models may lack the ability to describe the characteristics of the grain structure, such as its morphology and grain distribution. Cellular automaton (CA) has an intrinsic advantage over the description and visualization of microstructural evolution in metals and alloys [19,20]. It has been successfully applied to describe the evolution of the grain structure in alloy steels [21,22], titanium alloys [23,24,25], and Ni-based superalloys [26,27,28].

In this study, isothermal heat treatment experiments were conducted to investigate the grain growth behavior in a typical Ni-based superalloy. The grain structure of the studied superalloy was characterized by the electron backscatter diffraction (EBSD) technique. Compared with other characterization techniques, such as optical microscopy and scanning electron microscopy, the average grain size and grain size distribution can be readily and reliably measured by the EBSD technique. A cellular automaton model is developed to describe the evolution of grain structure in the studied Ni-based superalloy during isothermal heat treatment.

## 2. Materials and Experiments

A commercial Ni-based superalloy was used in this investigation. Table 1 shows its chemical composition.

Specimens with 5 mm × 5 mm × 2 mm dimensions were machined from the wrought billet. During the grain growth tests, specimens were heated in sealed quartz capsules together with high-purity argon gas. Ice water quench cooling was used to keep the microstructure of the specimen intact at the end of each heat treatment. Table 2 shows the detailed experimental procedure of grain growth tests. First, solution heat treatment was conducted for all specimens under a holding temperature of 1338 K for 1200 s. The solution heat treatment routine was carefully chosen to fully resolve all precipitates in the gamma matrix. Then, specimens were subjected to isothermal heating under a holding temperature of 1353–1473 K and holding time of 180–2400 s.

**Figure 1:**
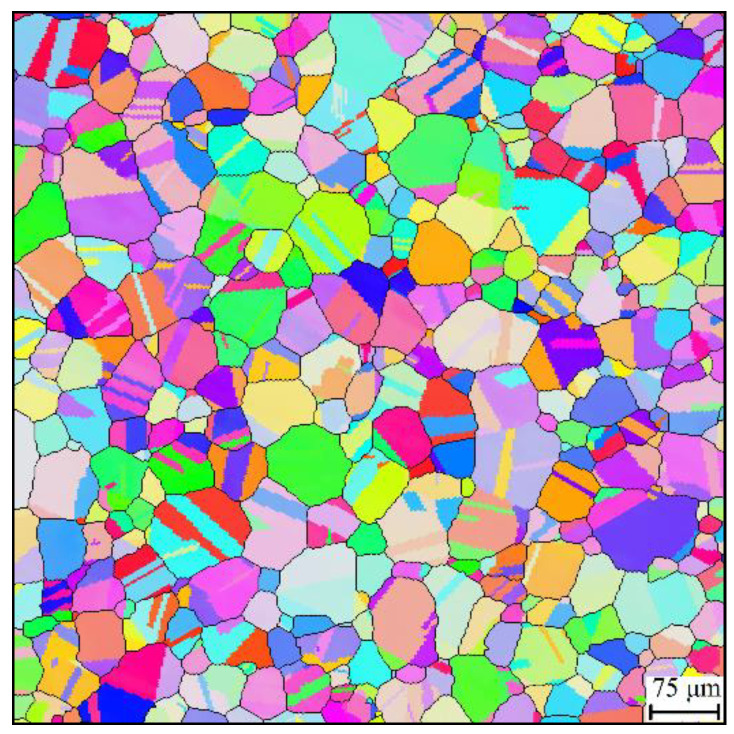
Initial grain structure for grain growth tests.

The microstructures of specimens after heat treatment were studied by electron backscatter diffraction (EBSD). Specimens were ground by sandpaper down to 1200 grit and final polished with a vibratory polisher with a colloidal silica suspension. The grain size and pH of the colloidal silica suspension were 0.04 μm and 9.8, respectively. EBSD detections were conducted on a Philips XL30FEG scanning electron microscope (SEM, FEI Co., Hillsboro, OR, USA), equipped with a charge-coupled device (CCD) camera. The detected EBSD data were processed with the texture analysis software MTEX version 5.7.0, Freiberg, Germany [29]. Figure 1 shows the grain structure after solution heat treatment. The grains are colored by inverse pole figure color keys [30]. The grain boundaries are represented by black lines. Twin grains sharing a common twin boundary were merged together for evaluation of the average grain size. Then, the average grain size was calculated by the area fraction weighted method [31]. The average grain size of the grain structure shown in Figure 1 is around 70 μm.

## 3. Experimental Results and Discussions

**Figure 2:**
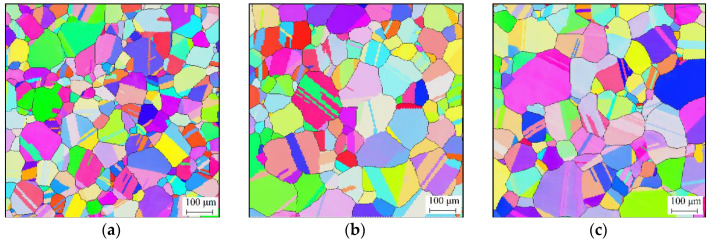
Grain structures under holding temperature of 1353 K and holding time of (**a**) 600 s, (**b**) 1500 s, and (**c**) 2400 s.

Figure 2 illustrates the effects of the holding time on the grain structure under a holding temperature of 1353 K. It is shown that the grain structure gradually coarsens as the holding time increases. The grain sizes under holding times of 600 s, 1500 s, and 2400 s were 106 μm, 154 μm, and 192 μm, respectively. During the isothermal holding period, the total grain boundary energy tends to reduce with increasing holding time [32,33]. Then, the reduction tendency of the grain boundary energy drives the grain boundaries to move and diminish, during which the average grain size grows with an increase in holding time.

**Figure 3:**
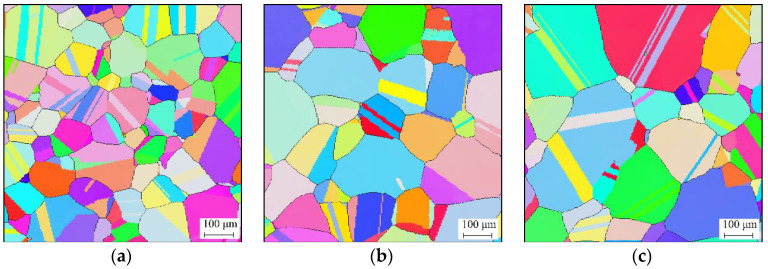
Grain structure under holding time of 540 s and holding temperature of (**a**) 1413 K, (**b**) 1443 K, and (**c**) 1473 K.

Figure 3 shows the effects of the holding temperature on the grain structure under a holding time of 540 s. It was found that the grains grew significantly under higher holding temperatures. The grain sizes under the holding temperatures of 1413 K, 1443 K, and 1473 K were 160 μm, 238 μm, and 299 μm, respectively. The movement of grain boundaries is a thermally activated process [34,35]. This makes the grain structure coarsen faster at higher holding temperatures.

**Figure 4:**
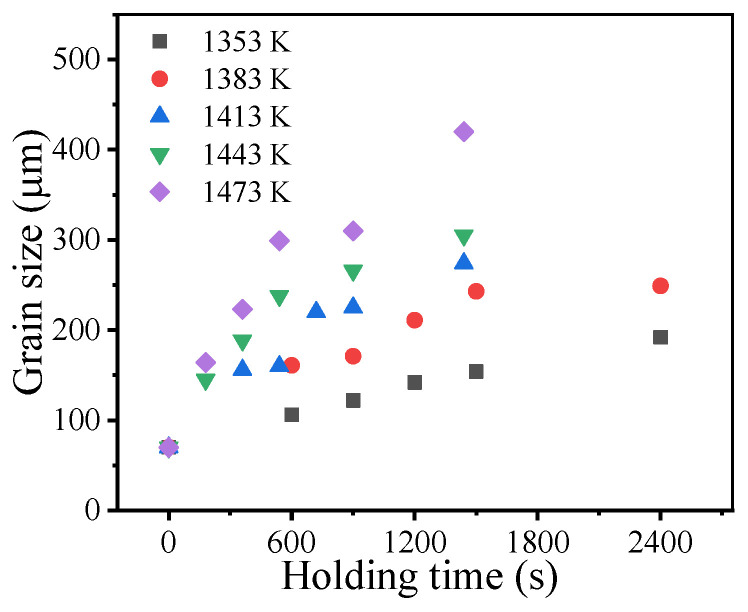
Grain sizes at different holding times and holding temperatures.

Figure 4 illustrates the measured grain sizes at different holding times and holding temperatures. The measured grain size continuously increases as the holding time and holding temperature increase. Generally, the relationship between the grain size and holding time during isothermal heat treatment can be described by the parabolic law [36]:

(1) $$G_{t}^{n}-G_{0}^{n}=k_{G}t$$

where n is the grain growth exponent, $G_{t}^{n}$ is the average grain size at time *t*, $G_{0}^{n}$ is the initial grain size, and $k_{G}$ is the material parameter and can be written in the Arrhenius form:

(2) $$k_{G}=k_{0}\exp[-Q/(RT)]$$

where $k_{0}$ is the material constant, Q is the activation energy, R is the gas constant, and *T* is the holding temperature.

Material parameters n, $k_{0}$, and Q can be identified by minimizing the value of the objective function (*OF*):

(3) $$OF=\sum_{j=1}^{\mathrm{nmd}}{\left(G_{m}^{j}-G_{p}^{j}\right)}^{2}$$

where nmd is the number of measured data, and $G_{m}^{j}$ and $G_{p}^{j}$ are the *j*th values of measured and predicted grain size, respectively.

Based on the MATLAB fminsearch function, n, $k_{0}$, and Q were identified by minimizing *OF*. Table 3 shows the identified material parameters. Thus, the relationship between the grain size and holding time during isothermal heat treatment for the studied superalloy can be described as:

(4) $$G_{t}^{2.43}-G_{0}^{2.43}=3.63\times 10^{14}\exp[-3.2\times 10^{5}/\left(RT\right)]t$$

**Figure 5:**
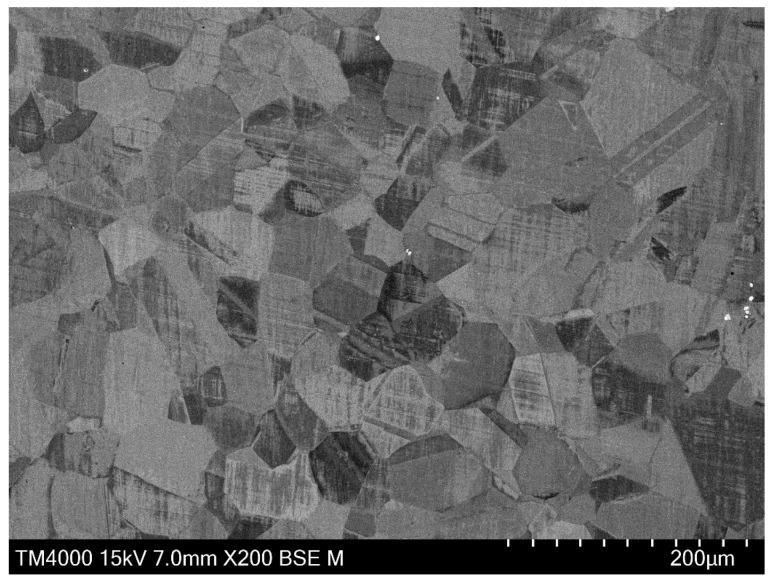
BSE image of the microstructure after solution heat treatment.

It was shown that the identified value of the grain growth exponent n was slightly above 2. The value of the grain growth exponent can be related with the grain growth mechanism and generally lies between 2 and 4 [36]. For a pure single-phase system, the grain growth exponent is theoretically equal to 2. The grain growth slows down in the presence of precipitates due to their suppressing effects [37,38]. Under these circumstances, the grain growth exponent increases. Thus, the identified grain growth exponent indicates that the current grain growth in the studied superalloy is mainly governed by grain boundary migration with a minor pinning effect from precipitates [39]. This is because most of the precipitates in the studied superalloy were dissolved during the solution heat treatment. Figure 5 shows the backscattered electron image of the microstructure after solution heat treatment. Except for some carbides, precipitates cannot be clearly seen. 

**Figure 6:**
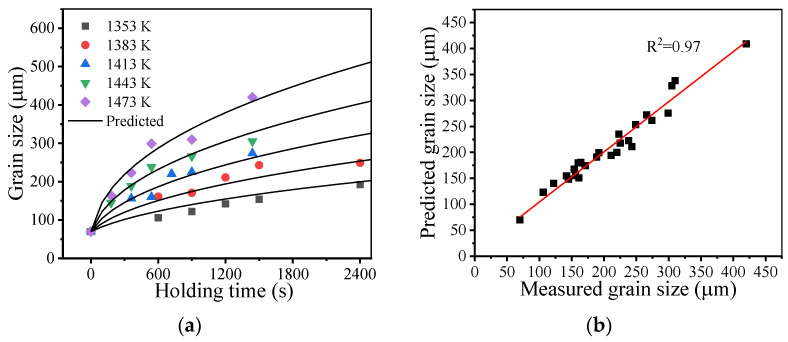
Comparisons between measured and predicted grain sizes: (**a**) fitting lines; (**b**) the coefficient of determination.

Figure 6 illustrates the comparison between the measured and predicted grain sizes. It was found that the predicted grain growth curves were in good agreement with the measured results. The coefficient of determination (R^2^) is 0.97. This suggests that Equation (4) can reliably capture the grain growth behavior of the studied superalloy.

## 4. Simulating Grain Growth Behavior Using a CA Model

A CA model is used to discretize the grain structure into a grid of cells and simulate the evolution of the grain structure by the transformation of cell states. The state of a cell is related with its neighboring cells according to the transformation rules. Thus, the grain growth can be described from a mesoscale view.

In this section, a CA model for describing the grain growth behavior of the studied superalloy will be established.

### 4.1. Models for Grain Growth

Grain boundary migration is mainly driven by the reduction of grain boundary energy. The moving velocity (*V*) of grain boundaries can be described by [40]:

(5) V=Mγκ

where *M* is grain boundary mobility, *γ* is grain boundary energy per unit area, and *κ* is grain boundary curvature.

*Γ* can be described by [41]:

(6) $$\gamma =\mu {b\theta }_{0}/\left[4\pi \left(1-\nu \right)\right]$$

where *μ* is the shear modulus for the studied superalloy, *μ* = *μ*_0_[1 − 0.64(*T* − 300)/1726], *μ*_0_ is the shear modulus at room temperature and is taken as 7.89 × 10^4^ Mpa, b is the magnitude of the Burgers vector, ν is the Poisson ratio, and θ_0_ is the lower misorientation limit for high angle grain boundary and is normally taken as 15°.

In reality, grain shape is never an ideal circle. Therefore, the grain boundary curvature cannot be explicitly evaluated by the circle area formula. After the grain structure is discretized into a grid of cells, the local grain boundary curvature at cell *i* can be evaluated by a template disk method [42,43]:

(7) $$\kappa ≃C_{\kappa }\left(N_{i}^{\kappa }-\mathrm{Kink}\right)/\left(D^{\kappa }N^{\kappa }\right)$$

where D*^κ^* is the diameter of the template disk, N*^κ^* is the total number of cells in the template disk, $N_{i}^{\kappa }$ is the number of cells with different states from the evaluated cell *i*, and Kink is the number of cells with different states from the evaluated cell *i* when the evaluated boundary is straight. In this way, $N_{i}^{\kappa }$ will be equal to Kink when cell *i* is located on a straight boundary. This makes Equation (7) equal to zero, which is consistent with the fact that the curvature of a straight boundary is zero. C*_κ_* is a parameter.

**Figure 7:**
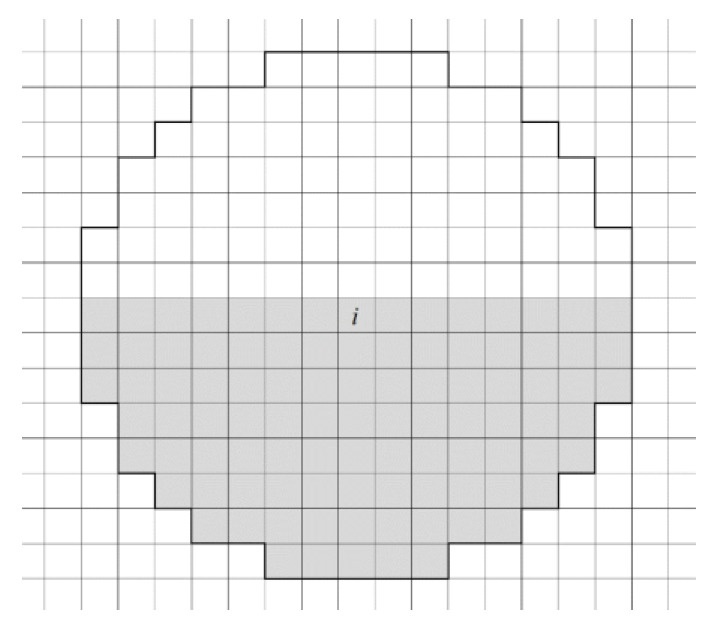
Template disk for evaluation of boundary curvature.

In the present study, a template disk with a diameter of 15 cells was employed to evaluate the boundary curvature at local cells [44]. By choosing an odd cell diameter, the local cell to be evaluated can be located right in the middle of the template disk. Figure 7 shows the detailed structure of the employed template disk. The template disk is surrounded by black lines. Within the template disk, there are 15 cells along the diameter and there are 177 cells in total. Thus, D*^κ^* = 15L_c_, N*^κ^* = 177, where L_c_ is cell size. To specify the value of Kink, Figure 7 also shows the details for straight boundary conditions. The colors white and gray represent different grains. Cell *i* is the cell where the boundary curvature will be evaluated and is located in the middle of the template disk. As shown in Figure 7, the local boundary at cell *i* is straight. Cell *i* belongs to the gray grain. There are 81 cells with a different state from the gray cells within the template disk. Thus, Kink = 81. From here, the curvature for the local boundary can be evaluated by moving the template disk along grain boundary cells, once C*_κ_* is determined.

C_*κ*_ can be calibrated by comparing analytical and simulated shrinkage results of one circular grain in terms of relationship between grain radius and time. The analytical equation describing the relationship between grain radius and time during the shrinkage of one circular grain can be written as:

(8) $$R_{t}^{2}=R_{0}^{2}-2M\gamma t$$

where *R_t_* and R_0_ are the grain radius at time *t* and the initial grain radius, respectively. For the calibration of C_κ_, one circular grain with an initial radius of 100 μm was chosen. *M* and *γ* are taken as the values of the studied superalloy at 1323 K [45]. The circular grain shrinkage simulation was conducted over a 200 × 200 square grid with a cell size of 2 μm. Cell neighbors were chosen according to Von Neumann’s neighboring rule and cell grid borders were treated by periodic boundary conditions [46]. The state transformation of a boundary cell is governed by a deterministic rule [47]. The moving distance of a boundary cell increases by:

(9) $$D^{t}=D^{t-\Delta t}+V\Delta t$$

where ∆*t* is the change in time. When *D^t^* becomes greater than the cell size, the state of the boundary cell transforms to the state of whichever neighboring cell has the largest curvature.

**Figure 8:**
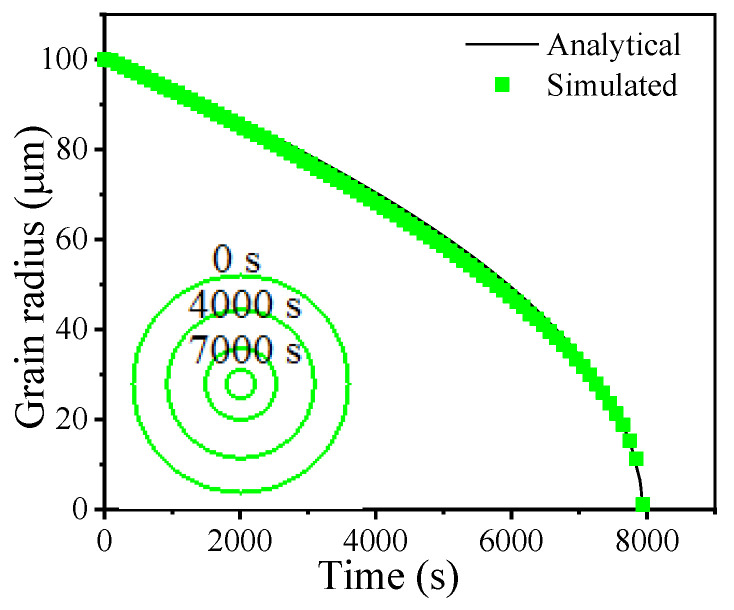
Comparison between analytical and simulated results for circular grain shrinkage.

Based on the analytical relationship between the grain radius and time, C_*κ*_ is calibrated as 17.6. Figure 8 shows the simulated relationship between grain radius and time after calibration. It was found that the simulated grain radius curve nearly coincided with the analytical one. The evolution of the grain shape with time during the shrinkage simulation is also shown in Figure 8. The grain shapes at 0 s, 4000 s, 7000 s, and 7800 s during simulation are indicated by green lines. The shrinking grain keeps a nearly circular shape even at the end of the simulation. This indicates that the current evaluation method for local boundary curvature is effective.

### 4.2. Grain Growth Simulation

A 500 × 500 square grid was used for the grain growth simulation. The grid was of the same cell size as the circular grain shrinkage simulation, and the transformation and neighboring rules and boundary conditions were also the same as in the circular grain shrinkage simulation. The initial microstructure for the simulation was created by having nuclei randomly seed in the 500 × 500 square grid and letting them grow until they impinged on each other. The initial grain size was controlled at 70 μm, which is the same as the measured value after solution heat treatment. Based on the measured grain sizes, the values of *M* under different holding temperatures were identified and listed in Table 4. It was found that *M* increases with increasing temperatures. This is because the movement of grain boundaries is a thermally activated process.

The relationship between *M* and holding temperature can be described by [48]:

(10) $$M={\delta D}_{\mathrm{ob}}b\exp\left[-Q_{b}/\left(RT\right)\right]/\left(kT\right)$$

where δD_ob_ and Q_b_ are material parameters, and R, b, and k are the universal gas constant, magnitude of Burgers vector, and Boltzmann’s constant, respectively. Based on the identified values of *M*, δD_ob_ and Q_b_ were fitted and are listed in Table 5.

**Figure 9:**
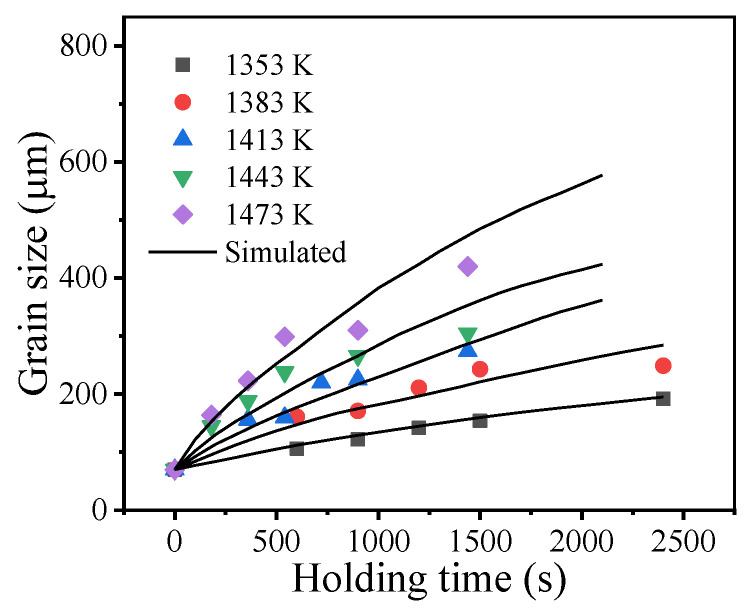
Comparisons between measured and simulated grain sizes.

**Figure 10:**
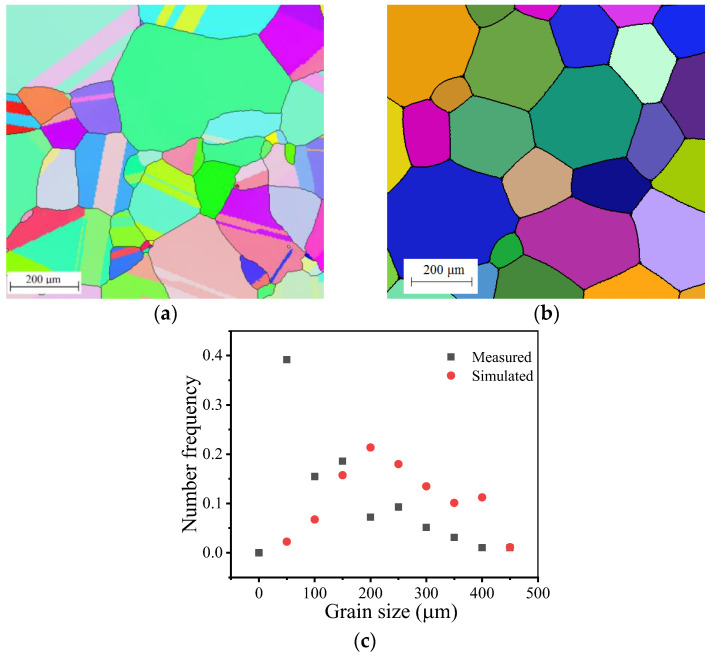
Comparisons between measured and simulated grain structure: (**a**) measured; (**b**) simulated; (**c**) grain number frequency.

Figure 9 shows the measured and simulated grain sizes under different holding temperatures. The simulated grain growth curves are capable of capturing the dependence of grain size on holding time under different holding temperatures. Figure 10 shows the comparisons between measured and simulated grain structure under the holding temperature of 1383 K and a holding time of 2400 s. The measured and simulated frequency patterns of grain size are similar. However, the measured frequency is much higher than the simulated value at a grain size of 50 μm and nearly all the simulated frequencies are higher than the measured values when the grain size is larger than 200 μm. These large differences in frequency pattern stem from a flaw in the initial microstructure creation method for CA simulation: during the creation of the initial microstructure for CA simulation, only the average grain size is controllable. The grain nuclei are randomly seeded, making the grain distribution uncontrollable.

## 5. Conclusions

The grain growth in the studied superalloy was investigated by isothermal heat treatments and a cellular automaton (CA) model. The following conclusions can be drawn:

(1) The holding temperature and holding time have large effects on the grain growth behavior of the studied superalloy. The grain structure gradually coarsens as the holding temperature and holding time increase.
(2) The grain growth behavior of the studied superalloy can be described by the parabolic equation $G_{t}^{2.43}-G_{0}^{2.43}=3.63\times 10^{14}\exp[-3.2\times 10^{5}/\left(RT\right)]t$.
(3) A CA model was developed to simulate the evolution of the grain structure in the studied superalloy. The local grain boundary curvature was effectively evaluated and the relationship between the grain boundary mobility and holding temperature was established. The simulated grain growth curves show that the developed CA model can capture the dependence of the grain size on the holding time under different holding temperatures.

## Figures and Tables

**Table 1:** Chemical composition (wt.%) of the studied superalloy.

Ni	Cr	Nb	Mo	Ti	Al	Co	C	Fe
53.82	18.38	5.42	3.03	0.95	0.41	0.2	0.027	Bal.

**Table 2:** Experimental procedure of grain growth tests.

Routine for Solution Heat Treatment	Holding Temperature for Grain Growth Experiments (K)	Holding Time for Grain Growth Experiments (s)
1338 K/1200 s + ice water quench	1353	600, 900, 1200, 1500, 2400
	1383	600, 900, 1200, 1500, 2400
	1413	360, 540, 720, 900, 1440
	1443	180, 360, 540, 900, 1440
	1473	180, 360, 540, 900, 1440

**Table 3:** Material parameters related with the prediction of grain size.

Parameters	n	$k_{0}(\mu m^{n}\cdot s^{-1})$	Q (kJ·mol^−1^)
values	2.43	3.63 × 10^14^	320

**Table 4:** Values of M for the studied superalloy.

**Temperature (K)**	1353	1383	1413	1443	1473
**M (m^4^ J^−1^ s^−1^)**	8.5 × 10^−12^	1.75 × 10^−11^	2.7 × 10^−11^	4 × 10^−11^	6.5 × 10^−11^

**Table 5:** Material parameters related with the calculation of grain boundary mobility.

**Parameter**	δD_ob_ (m^3^·s^−1^)	Q_b_ (J·mol^−1^)	R (J·mol^−1^·K^−1^)	b (m)	k (J·K^−1^)
**Value**	5.76 × 10^−12^	2.55 × 10^5^	8.314	2.49 × 10^−10^	1.38 × 10^−23^

## Data Availability

The data presented in this study are available in this article.
